# Brazilian version of Calgary-Cambridge Observation Guide 28-item version: cross-cultural adaptation and psychometric properties

**DOI:** 10.6061/clinics/2021/e1706

**Published:** 2021-06-07

**Authors:** Marcela C. Dohms, Carlos Fernando Collares, Iolanda Calvo Tiberio

**Affiliations:** I Clinica Medica, Hospital das Clinicas HCFMUSP, Faculdade de Medicina, Universidade de Sao Paulo, Sao Paulo, SP, BR; II Department of Educational Development and Research, School of Health Professions Education (SHE), Faculty of Health, Medicine and Life Sciences, Maastricht University, Maastricht, The Netherlands

**Keywords:** Communication Skills, Assessment, Medical Education, Primary Care, Psychometrics, Reliability

## Abstract

**OBJECTIVES:**

The search for appropriate tools to assess communicational skills remains an ongoing challenge. The Calgary-Cambridge Observation Guide (CCOG) 28-item version can measure and compare performance in communication skills training. Our goal was to adapt this version of the CCOG for the Brazilian cultural context and perform a psychometric quality analysis of the instrument.

**METHODS:**

Experienced preceptors (35) assessed videos of five medical residents with a simulated patient using the translated guide. For the cultural adaptation, we followed the methodological norms on synthesis, retro-translation, committee review, and testing. We obtained validity evidence for the CCOG 28-item version using confirmatory factor analysis and the Many-Facet Rasch Model (MFRM).

**RESULTS:**

Confirmatory factor analysis indicated an adequate level of goodness-of-fit. The MFRM reliability coefficient was high in all facets, namely assessors (0.90), stations (0.99), and items (0.98). The assessors had greater difficulty with attitudinal items, such as demonstration of respect, confidence, and empathy.

**CONCLUSIONS:**

The psychometric indicators of the tool were adequate, a good potential for reproducing its Brazilian version as well as acceptable reliability for its use.

## INTRODUCTION

Communication skills are crucial in medical practice. One contemporary challenge is to find the appropriate tools to assess these skills in medical education. The most used tools for evaluating communication skills have been developed as a checklist for observation, usually used in summative assessments in Objective Structured Clinical Examination-type (OSCE-type) for performance comparisons and formative feedback (1,2).

We opted to use the Calgary-Cambridge Observation Guide (CCOG) 28-item version, an evaluation instrument derived from Calgary-Cambridge Process Guides. The guide was first published in 1996 in Canada by the same authors with 71 items, for use as an observation guide during teaching medical interviews. The 28-item version was developed for a different proposal: to assess history-taking interviews in OSCE-type stations, with a questionnaire divided into six blocks/domains, according to its several stages, with a 3-point scale (“Yes”, “Yes, but” and “No”) in a checklist format. This version presented adequate psychometric properties in previous studies (1).

Our goal was to develop a translation and cross-cultural adaptation to Brazilian Portuguese of the 28-item CCOG questionnaire to assess medical communication skills as well as to analyze the psychometric quality of the instrument and present our preliminary validation results.

## MATERIALS AND METHODS

We adopted methodological norms recommended by researches, which contained the following stages: translation; synthesis; retro-translation; review by a committee; and pre-testing. Initially, two bilingual translators (native Brazilians) performed two independent translations from English to Portuguese-BR. The instrument was then sent to an expert committee, consisting of six medical educators with teaching experience in communication skills. The committee validated the content, analyzed the differences on each translated item using the online platform Google Forms, and provided additional suggestions. Subsequently, all versions of the tool were merged and reviewed into the pre-test version. After reaching a consensus, we considered the semantic, idiomatic, experimental, and conceptual equivalences. Following a review of the suggestions, we developed a pre-test version and applied the tool to the research participants, 35 preceptors in a Primary Care program.

Initially, we instructed all participants on filling out the questionnaire and discussed their doubts and concerns. Subsequently, they watched the five videos in which five doctor-residents performed the same OSCE station with a simulated patient. Each video had a maximum running time of seven minutes, after which they answered the 28-item CCOG questionnaire for each video. Once the questionnaires were completed, we discussed their understanding of each item and the difficulties encountered in the process. We took notes of their suggestions for altering any item, which we later discussed among the authors for modifications in the final version. The translated final version is available in the Appendix.

We estimated the intra-class correlation coefficient (ICC) for evaluating the inter-rater reliability for each domain of the questionnaire. Furthermore, we estimated the Cronbach's alpha coefficient to assess the internal consistency of each domain. To evaluate the degree of importance of each question in each domain, we calculated the Cronbach's alpha coefficient with the exclusion of each question. We evaluated the correlation between the domains of the questionnaire by estimating the Spearman correlation coefficient and testing its significance. Values of *p*<0.05 indicated statistical significance. We then analyzed the data by using the IBM SPSS Statistics v.20 software.

We also analyzed the data by using the Many-Facet Rasch Model (MFRM), developed by Linacre (3), which provides additional evidence to validate the interpretation of the scores. For measuring the psychometric quality of the tool in this analytical model, we analyzed how multiple variables may simultaneously influence the scores, allowing us to estimate a completely neutral examiner and reach an estimated “fair score” (4). The MFRM model has been increasingly used for analyzing the quality of assessments with response items. It allows us to include other important variables that may be bias generators in assessment processes, such as the personal characteristics of the assessors, their propensities, and criteria differences regarding severity or understanding (5). When we observe the adequacy to the MFRM model it means we have attained invariance measures, which implies that particular items did not influence the measurements of the persons. Furthermore, the measurement of the items was not affected by the variance in assessors (6). The model attempts to calibrate items regardless of the persons involved (7 [Bibr B08]). We analyzed the data with the FACETS software version 3.71.4 to run the MFRM model and Mplus version 8 to run the confirmatory factor analysis.

### Ethics approval and consent to participate

The Ethics Committee of the Municipal Health Secretariat of Rio de Janeiro (CAAE: 57387816.7.0000.5279) approved this research project. All participants signed a Free and Informed Consent Form with clarifications about the research.

## RESULTS

### Descriptive statistics and inter-rater agreement

Table 1 (below) shows that the item with the highest agreement among assessors was item 23 (“Demonstrates no prejudice or judgment”). The items with the least agreement were 4 (“Identifies and confirms problems list”), 12 (“Establishes dates and sequence of events”), and 28 (“Contracts with the patient the next steps”). In many items, we found a similar percentage between “Yes” and “Yes, but...”.

As shown in Table 2 below, the domains with the best agreement between assessors, as demonstrated by intraclass correlation coefficient (ICC) were 2 (“Exploring problems”) and 3 (“Assessment of the patient’s representations”), while the worst was 6 (“Concluding the consultation”).

### Confirmatory factor analysis

The results of the confirmatory factor analysis indicate an acceptable level of fit of the Brazilian version of the 28-item CCOG. The χ^2^/df ratio was 1.92, below the desirable thresholds recommended in the literature (9). The CFI, which is the comparative adjustment index, corresponds to the best adjustment of the data model when the variables are independent. The observed value (0.90) was slightly above the generally acceptable threshold (0.90) (10). When we exclude items 1 and 2 due to their lack of variance, the value rises to 0.91. The TLI (Tucker-Lewis Index) ranges from 0 to 1, with 1 referring to a perfect fit. Its value was below the threshold (0.90) if all items are taken into account (0.84) and borderline when items 1 and 2 are excluded (0.90) (11). The RMSEA (Root Mean Square Error of Approximation), which reflects the average difference between the observed covariance and the model, was 0.07 and presented a value within the desirable threshold, which is up to 0.08 (11). The WRMR index was 1.21, slightly above the desirable limit of 1.0 (10).

### Many-Facet Rasch Model

**Figure 1:**
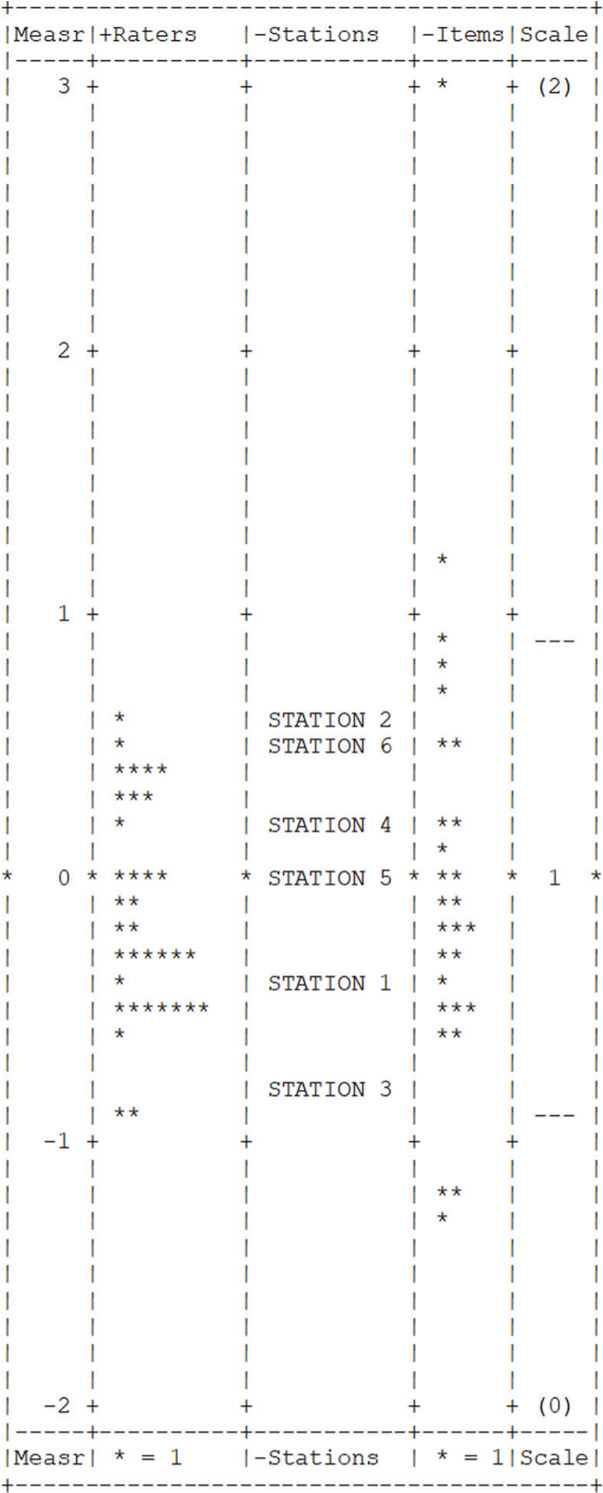
Yardstick graph with the distribution of parameters from the MFRM analysis.

MFRM analysis resulted in high reliability coefficients for all facets: raters (0.90), stations (0.99), and items (0.98). Infit and outfit measures were in the acceptable range (0.5-1.5) for all items, with the exception of item 1 (*infit*=1.67). Figure 1 shows a Yardstick graph with the distribution of the estimated parameters according to the MFRM analysis.

## DISCUSSION

The study of the cultural adaptation process of the internationally validated CCOG 28-item version questionnaire showed good results for this Brazilian sample. Reliability estimates have surpassed Streiner’s suggestion (8) that reliability coefficients should be between 0.80 and 0.90, which suggests that the Brazilian version has an acceptable level of reliability, even though this study has used the MFRM psychometric approach instead of more traditional internal consistency coefficients, such as Cronbach’s alpha.

Items 1 (“Greets patient”) and 2 (“Introduces self and role”) of the tool showed negative loads in the confirmatory factor analysis, suggesting that these are not adequately measuring the intended construct. The analysis indicated that the intended construct may explain the 0% variance of these two items as 100% noise or another non-intended construct. The determination coefficient (R^2^) indicates to what extent a variation of a variable can be explained by another variable (in our case, the item verse construct). It also had a low value (zero), possibly because it was a simulated station, and the recording began inside the office. However, some resident doctors greeted the patient before entering the consultation and began filming, thus interfering with the analysis. Since the instructions were to leave a blank if the task could not be assessed, these initial items for starting the consultation had the highest number of blanks.

We see an improvement in the model adjustment if we repeat the same type of analysis without items 1 and 2. The adjustment indices about the proposed theoretical model and the significant increase of the adjustment obtained with it compared to a one-dimensional model are validity evidence based on the internal structure of the tool. For analyzing items 1 and 2, we suggested that the consultation should start in the videotaped environment so that we may observe the interviewer greeting and introducing themselves to the patients.

The item with the highest agreement among assessors was that medical residents did not show their judgment. It seems to be a clearer parameter, and one that residents are usually well trained to avoid. An item with two tasks had the highest disagreements among assessors: “Identify and confirm problems list”. Perhaps having two tasks on the same item interfered with the variation in responses. We, therefore, suggest changing this item to “Confirm the list of problems”, since to confirm the problems, the interviewer must have already identified them. Furthermore, we observed that the item “Negotiates agenda” demands further attention when dealing with students at the beginning of the medical course, since they may find it harder to address multiple topics when learning how to collect the patient's history.

We found significant disagreements in the item “Establishes dates”, which impelled the assessors to suggest changes. The difficulty in understanding the meaning of the task was the most likely culprit in the divergence of answers, and we modified the final version accordingly. The last item of the questionnaire, regarding the ability to make a shared decision, also showed a high degree of disagreement, possibly because a complete agreement with the patient involves a complexity of dialogs and negotiations, which may require better-defined parameters. There was a low intra-class correlation coefficient in the domain “Concludes the consultation”, probably because of the difficulty in understanding the shared decision-making process in the item “Contracts with the patient the next steps.” We believe that the word “contract” may give leeway to different interpretations as to what one considers a satisfactory degree of patient participation in the decision-making process.

We observed other items with a more subjective interpretation, which led to further significant differences in the assessment. The assessors mentioned difficulties when defining parameters in less objective or technical behavioral assessment items such as “Demonstrates respect / appears confident / demonstrates empathy.” We believe these items need further development when defining their parameters among assessors in light of the learning objectives in each phase of the medical education. Moreover, external observers may find it challenging to judge complex tasks. For a complete assessment, we would need to know the patient's opinion, such as if the interviewer conveyed confidence or empathy. The group of assessors must discuss these items in further detail to define what they consider to be satisfactory, unsatisfactory, or partially satisfactory.

We observed that when an item was not performed, the assessors found it easier to check “No.” However, when the residents performed the task, the assessors were often in doubt between “Yes” and “Yes, but...”, thus indicating the need for a better definition of when a task is wholly or partially accomplished. These difficulties may have interfered in some of the tool’s reliability and validity coefficients. Furthermore, we attribute the reliability difficulties of the scale in the study to the need for further instructions and better-defined parameters among assessors before the application (12). Nonetheless, another study about scale-validation in Germany showed similar intra-class correlation coefficients ranging from 0.05 to 0.57.

The difficulties observed when assessing and judging the items may affect the final summative assessment. Considering this, a subjective holistic judgment may be beneficial (13). Studies that compared the psychometric properties of checklists and global assessment scales in OSCEs assessed by experts suggested the superior validity of global assessment over checklists (14,15). In its original version, the tool predicts a global evaluation with no note value between “Satisfactory”, “Satisfactory, but...” and “Unsatisfactory”, which we did not use in the study. Nevertheless, we recommend its regular use alongside the questionnaire.

The CCOG 28-item version tool may be used for both formative and summative assessments. Due to the aforementioned difficulties in interpreting more subjective items, we believe that the instrument may be more beneficial when applied to a formative assessment. We suggest the inclusion of narrative feedback when using the tool in a summative assessment, as students not only appreciate them, but the effectiveness of this method has proven to be high (16).

Despite the difficulties observed in the study, the reliability coefficients in the Many-Facet Rasch Model were excellent across all facets. High reliability estimates lower the risk of false positives or false negatives in assessment. A lower measurement error demonstrates that the tool has acceptable reliability for reproducibility in other contexts. One limitation of this study was the diverse background of the assessors and experienced preceptors for assessing communication skills. Additionally, a larger sample could provide us with more information. Another limitation was that we were unable to confirm the reliability of the assessment among each assessor in a second one. Furthermore, the preceptors' assessment could have been associated and compared to evaluations from other sources such as colleagues, staff, and simulated-patients, considering that multiple-source assessors in the Medical Residency Programs can qualify assessments of attitudinal skills and complex tasks (17,18).

While we designed the study with resident physicians, we believe that the CCOG 28-item version could also be used with undergraduate students, as previously demonstrated in other studies, with the standardization of the parameters of items according to the course period and learning objectives (12). We underline the importance of discussing with the group of assessors the meaning of each word in the questionnaire, just as its subsequent practical use is essential for constant improvements, which should undergo further adjustments with the feedback. We suggest additional researches on assessment tools for medical communication, with a better definition of subjective items according to the learning objectives.

The validity of an instrument is a continuous process (19,20) and the questionnaire must be continually reevaluated for improvements. We also emphasize the importance of homogenizing the assessment parameters among assessors on each item before applying the tool, as well as clarifying the learning objectives required for each training level. This becomes particularly important when assessing demonstration of respect, confidence, and empathy, which are less objective attitudinal assessment items. We also suggest complementing the medical communication assessment with other viewpoints such as those of colleagues, patients, and staff.

## CONCLUSIONS

The reliability indicators of the MFRM suggest reasonable reproducibility and stability of the assessors should they need to evaluate the same people at a different time. The Brazilian translation of the CCOG 28-item version had acceptable reliability in assessing communication skills and it may be an adequate tool in the systematic assessment of communication skills in Brazil, as currently used in other countries. We encountered limitations regarding assessors and sample size. We recommend more detailed instructions and better-defined parameters for the assessors before applying the instrument, as well a complementary overall evaluation. We also suggest associating the scale with detailed narrative feedback in formative assessment and a continual reevaluation of the tool for constant improvements (21).

## AUTHOR CONTRIBUTIONS

Dohms MC was responsible for all facets of the study, from study design to data collection and analysis to completion of the manuscript. Collares CF was responsible for the study conception and development, data interpretation, manuscript revision and editing. Tiberio IC was responsible for the study conception, development and design, data interpretation, and manuscript review. All of the authors have read and approved the final version of the manuscript.

## Figures and Tables

**Table 1:** Descriptive statistics of responses to the 28-item CCOG questionnaire with percentages according to response options.

		Analyzed video				
Questionnaire Item	Assessors' response	1	2	3	4	5
1. Greets patient.	No	0	76	74	6	18
	Yes, but...	17	21	7	9	12
	Yes	83	31	19	85	69
2. Introduces self and role.	No	83	94	96	94	100
	Yes, but...	11	6	4	6	0
	Yes	6	0	0	0	0
3. Demonstrates respect.	No	0	13	3	0	6
	Yes, but...	57	77	6	23	42
	Yes	43	9	91	77	52
4. Identifies and confirms problems list.	No	27	50	21	40	22
	Yes, but...	48	37	29	40	53
	Yes	24	13	50	20	25
5. Negotiates agenda (reasons for consultation).	No	57	92	48	75	63
	Yes, but...	36	8	24	21	18
	Yes	7	0	28	4	18
6. Encourages the patient to tell story.	No	26	42	0	9	10
	Yes, but...	47	37	24	29	48
	Yes	27	21	76	62	42
7. Appropriately moves from open to closed questions.	No	53	76	15	53	42
	Yes, but...	30	15	37	29	52
	Yes	17	9	47	18	6
8. Listens attentively.	No	20	41	3	3	9
	Yes, but...	48	50	12	44	56
	Yes	31	9	85	53	34
9. Facilitates patient’s responses verbally and non-verbally.	No	35	83	0	38	36
	Yes, but...	35	17	33	38	61
	Yes	29	0	67	24	3
10. Uses easily understood questions and comments.	No	3	12	0	9	10
	Yes, but...	21	39	9	61	50
	Yes	76	49	91	30	40
11. Clarifies patient's statements.	No	10	22	7	39	22
	Yes, but...	45	61	36	50	55
	Yes	45	16	57	11	22
12. Establishes dates and sequence of events.	No	43	50	17	37	21
	Yes, but...	29	32	27	33	27
	Yes	28	18	56	30	52
13. Determines and acknowledges the patient's ideas regarding cause.	No	23	12	12	52	10
	Yes, but...	17	72	24	33	51
	Yes	60	16	64	15	39
14. Explores patient's concerns regarding the problem.	No	6	13	15	47	12
	Yes, but...	17	56	21	37	41
	Yes	77	31	64	16	47
15. Encourages the patients to verbalize how they feel.	No	42	65	28	81	52
	Yes, but...	23	31	28	19	28
	Yes	35	4	44	0	20
16. Picks up/responds to verbal and non-verbal clues.	No	43	78	42	79	42
	Yes, but...	48	22	35	21	42
	Yes	9	0	23	0	16
17. Summarizes at end of a specific line of inquiry.	No	32	62	31	50	39
	Yes, but...	36	31	31	28	42
	Yes	32	7	38	22	19
18. Progresses using transitional statements.	No	46	68	38	70	58
	Yes, but...	36	28	23	23	32
	Yes	18	28	39	7	10
19. Structures logical sequence.	No	9	29	21	73	27
	Yes, but...	32	32	21	12	52
	Yes	59	39	58	15	21
20. Uses time efficiently.	No	0	21	6	61	41
	Yes, but...	20	46	23	29	24
	Yes	80	32	71	10	35
21. Demonstrates appropriate non-verbal behavior.	No	38	70	3	38	35
	Yes, but...	32	18	18	31	55
	Yes	55	12	79	31	10
22. If reads or writes, does so without interfering with dialogue/rapport.	No	8	80	4	70	58
	Yes, but...	23	1	11	18	24
	Yes	69	7	85	12	18
23. Demonstrates no prejudice or judgment.	No	11	4	0	11	19
	Yes, but...	21	29	11	26	23
	Yes	68	67	89	63	58
24. Demonstrates empathy and supports patient.	No	3	24	6	17	16
	Yes, but...	43	65	45	62	68
	Yes	54	10	49	21	16
25. Appears confident.	No	3	25	0	49	28
	Yes, but...	38	53	15	27	53
	Yes	59	22	85	24	19
26. Encourages patient to discuss additional issues.	No	40	50	78	33	52
	Yes, but...	24	28	19	37	29
	Yes	36	22	3	30	19
27. Concludes consultation with a brief summary.	No	47	63	35	24	27
	Yes, but...	27	25	24	46	30
	Yes	26	12	41	30	43
28. Contracts with the patient the next steps.	No	41	55	12	23	16
	Yes, but...	28	35	36	42	34
	Yes	31	10	52	35	50

**Table 2:** Intraclass correlation coefficients (ICC) for each domain (in percentage).

Domain	Theme	ICC
1	Beginning the consultation.	36.2 %
2	Exploring problems.	45.8 %
3	Understanding the patient’s perspective.	27.7 %
4	Structuring the consultation.	32.1 %
5	Building the relationship.	45.3 %
6	Concluding the consultation.	6.8 %

## APPENDIX

**Table:** Brazilian version of CALGARY-CAMBRIDGE OBSERVATION GUIDE 28-item.

INICIANDO A CONSULTA	Não (0)	Sim, mas (1)	Sim (2)
1. Cumprimenta o paciente.			
2. Apresenta-se e menciona a sua função.			
3. Demonstra respeito.			
4. Confirma os motivos de consulta.			
5. Negocia a agenda (motivos de consulta).			
**OBTENDO INFORMAÇÕES** ***Exploração dos problemas***			
6. Encoraja o paciente a contar sua história.			
7. Muda apropriadamente de questões abertas para fechadas.			
8. Escuta atentamente.			
9. Facilita respostas verbais e não verbais do paciente.			
10. Utiliza perguntas e comentários facilmente compreensíveis.			
11. Esclarece as declarações do paciente.			
12. Define cronologia dos problemas.			
***Compreendendo a perspectiva do paciente***			
13. Determina e reconhece as ideias do paciente sobre a causa do problema.			
14. Explora as preocupações do paciente sobre o problema.			
15. Estimula que o paciente verbalize como se sente.			
16. Percebe e responde às pistas verbais e não-verbais.			
***Estruturando a consulta***			
17. Resume ao final de uma linha específica de investigação.			
18. Progride usando frases de transição entre os tópicos.			
19. Estrutura uma sequência lógica.			
20. Usa o tempo de maneira eficiente.			
***Construindo a relação***			
21. Demonstra comportamento não verbal apropriado.			
22. Se lê ou escreve, isto não interfere com o diálogo/comunicação.			
23. Não demonstra preconceito ou julgamento.			
24. Demonstra empatia e apoio ao paciente.			
25. Demonstra confiança.			
**ENCERRANDO A CONSULTA**			
26. Encoraja o paciente a discutir mais algum ponto adicional.			
27. Encerra a consulta com um breve resumo.			
28. Pactua com o paciente os próximos passos.			

Based on Kurtz et al. (1).

(Tradução e adaptação transcultural com permissão da autora Suzanne Kurtz.)
